# Should Teachers Carry Guns? An Emergency Room Survey of Parents of Two New York Communities

**DOI:** 10.7759/cureus.34962

**Published:** 2023-02-14

**Authors:** Muhammad Waseem, Kirsten Morrissey, Ashley Nelsen, Ashar Ata, Hina Asad

**Affiliations:** 1 Department of Emergency Medicine, New York City (NYC) Health and Hospitals Lincoln Medical Center, New York City, USA; 2 Department of Emergency Medicine, Albany Medical Center, Albany, USA; 3 Department of Surgery, Albany Medical Center, Albany, USA

**Keywords:** firearms, school shooting, emergency department, child safety, gun violence

## Abstract

Introduction: The purpose of this study was to compare parents’ perceptions of threats and solutions to school gun violence in two different communities.

Methods: Parents of school-aged children visiting emergency rooms of two large trauma centers in Upstate New York (UNY) and New York City (NYC), between October 2019 and December 2020, were surveyed (UNY: n=202, NYC: n=100). Responses were compared by site, firearm experience, and concern for school safety.

Results: Respondents from the two sites differed by sociodemographic characteristics. Of the 302 respondents, 64% feared a school shooting incident, but UNY respondents were less likely to report concern (46.5% vs 99%, p<0.001). UNY respondents were more likely to feel safe for their children (75.3% vs 7%, p<0.001) and to report feeling safer if guns were available to teachers (22.3% vs 6%, p <0.001). Both sites’ respondents agreed on the need for armed police presence (76.7% vs 74%, p=0.11). Of the 193 parents concerned about a school shooting, 11.9% indicated feeling safer if guns were available to teachers versus 25.7% of those who were not (p=0.002). Agreement on solutions for making schools safer differed by the site. NYC respondents were unanimously supportive, but UNY support ranged from 52% for metal detectors to 84.5% for controlled entry points.

Conclusion: Although perceptions of child safety and experience with guns varied by location, most parents agreed on potential solutions, that it should be the security officers, not teachers, who should be carrying firearms and that armed police should be present in schools to provide safety.

## Introduction

Firearm-related deaths are a leading cause of death in US children, surpassing the number of deaths from pediatric congenital anomalies, heart disease, influenza, pneumonia, chronic lower respiratory disease, and cerebrovascular causes [1]. Firearms have been associated with more than 100,000 injuries and about 40,000 deaths annually in the US [2]. In the US, school-aged children are more likely to die due to a firearm injury than from any other single cause of death [3]. As compared with other developed countries, in the US children between the ages of 5 and 14 years are 21 times more likely to be killed by guns, and those between the ages of 15 and 24 years are 23 times more likely to be killed by guns [4]. Based on data from 2012 to 2014 approximately 5,790 children each year are treated in emergency departments (ED) in the US for a firearm-related injury and up to 1,300 American children are killed by guns every year [5]. For gun violence, in general, the year 2020 emerged as the deadliest year in two decades and the year 2021 showed very early signs of exceeding 2020 [6]. As of December 20th, 2021, there have been 34 school shootings that year, resulting in 11 student deaths, three staff or employee deaths, and 54 people injured [7]. The widespread publicity of mass shootings, including school shootings, has raised a question in the public as to the best way to make schools safer and to help deter school shootings. One solution that has been raised in the public is the possibility of arming teachers and/or school staff. This sparked a controversy about whether arming teachers with guns would make schools safer.

Although the idea of arming teachers has continued to be controversial, schools across the nation have recognized the need for safer schools and have implemented a variety of strategies to increase safety such as installing metal detectors, hiring security guards, or installing video cameras around the buildings. Many schools are “gun-free zones,” but the federal Gun-Free School Zones Act allows states to authorize certain individuals to carry firearms on school grounds. For example, states like Texas, Utah, Wyoming, and South Dakota allow staff members to carry guns on school grounds, with some qualifications [8]. In general, states with more permissive gun laws and greater gun ownership have had higher rates of mass shootings [9].

By surveying parents of school-aged children, we sought to better understand parental opinions on arming teachers in schools as a deterrent to school firearm violence, as well as parental perceptions of school safety. We also examined parents’ opinions on alternative measures to improve personal safety in schools.

## Materials and methods

Following a literature review and an assessment of community needs a survey was developed by the authors with the goal to determine parental opinion regarding the arming of teachers in schools. Questions were developed utilizing a combination of multiple-choice questions using a 5-point Likert scale, open-ended questions, as well as questions with options for multiple responses. Demographic data were collected including parent age, gender, race, education, and location. The survey contained questions regarding parental perceptions of school safety, experience with both firearms and violence, and potential solutions. These included arming school staff, methods for increasing security in schools, addition of metal detectors, increased education, and, hiring of security officers. Parents also had the opportunity to further explain their answers and to write additional solutions or concerns. The study was approved by the Institutional Review Boards (IRB) at each institution. We also administered the survey questionnaire to the parents of school-aged children, who visited the ED of two level-I large trauma centers in New York State. These centers represent large trauma centers in Upstate New York (UNY) and New York City (NYC). This study was conducted between October 2019 and December 2020.

Inclusion criteria comprised surveys of both the biological parents or legal guardians of school-aged children (defined as ages 5th until 18th birthday) during an ED visit by either a child or a visit by the parent. Subjects were excluded if children triaged as critical and require immediate intervention or resuscitation, or parent presenting with an altered mental status or other inability to complete the survey. Children who were not accompanied by their biological parents or primary guardians at the time of the ED visit were also excluded. Parents were surveyed either in the pediatric or in the adult EDs. Potential subjects were consented to and enrolled via convenience sample based upon the availability of research staff. At UNY, we used a locally developed system for random sampling called the Time-Refreshed Electronic Tracker Sampling (TRETS), which is a previously validated random sampling technique derived from the time length-of-stay times on the electronic patient tracker. This system uses the electronic ED tracker to randomly sample an ED patient who has been in ED for 1 to 4 hours. Data was collected at UNY between October 1, 2019, and January 31, 2019. At the UNY location, 210 surveys were initiated, and eight were ultimately excluded for incomplete or limited data collection. Data in NYC was based on a convenience sample collected between January 2020 and December 2020. Responses were compared by ED site, and by parents’ experience with firearms and concern for school safety employing t-tests and Chi-square and Fisher’s Exact tests, as appropriate. Statistical software STATA 15.1 was used for analysis.

## Results

Demographics

A total of 302 adult patients with school-aged children or parents of school-aged patients completed the survey, including 202 from UNY and 100 from NYC. There was a significant difference in the sociodemographic composition of the respondents from the two study sites (Table 1). The respondents from UNY were older than those from NYC (mean: 40 years, SD: 8.3 vs mean: 34.0 years, SD: 8.3 respectively, p<0.001). About three-fourths of the respondents from the UNY site were females compared to about three-fifths of the NYC respondents (p=0.003). A majority of UNY (67.5%) respondents were Caucasian/White and the majority (81%) of NYC respondents were Hispanic or Latino (p <0.001). There was a significant difference in the education levels of the respondents from the two places where 75.6% of UNY and 98% of the NYC respondents had a college education or less (p<0.001), respectively. Only about a quarter (26.7%) of UNY respondents reported living in an urban environment as compared to 100% of respondents in NYC (p<0.001).

Table 1 further describes the demographic characteristics while Table 2 compares parental responses concerning their perceptions of safety at school, potential solutions, and their experiences with guns.

**Table 1:** Demographic characteristics

	Upstate New York (N=202)	New York City (N=100)	
Demographic characteristics			
	n (%)	n (%)	P value
ED Location			<0.001
Adult	41 (20.30)	0 (0)	
Pediatric	161 (79.70)	100 (100)	
ED Status			<0.001
Parent of patient	161 (79.70)	100 (100)	
Parents of children in adult ED	41 (20.30)	0 (0)	
Age, mean (SD), years	40 (8.26)	34 (5.79)	<0.001
Sex			0.003
Male	49 (24.38)	41 (41)	
Female	152 (75.62)	59 (59)	
Race			<0.001
Caucasian/White	135 (67.50)	0 (0)	
Hispanic or Latino	15 (7.50)	81 (81)	
Black/African American	24 (12)	17 (17)	
Asian/Pacific Islander	11 (5.50)	2 (2)	
Other	15 (7.50)	0 (0)	
Education			<0.001
College or less	152 (75.62)	98 (98)	
Master's, Professional or Doctoral Degree	49 (24.38)	2 (2)	
Living Location			<0.001
Urban	51 (26.70)	100 (100)	
Suburban	92 (48.17)	0 (0)	
Rural	48 (25.13)	0 (0)	

**Table 2:** Parents’ responses concerning their perceptions of safety at school, potential solutions, and their experiences with guns

Survey Responses	n(%)	n(%)	P value
How safe do you feel that your child is in school?			<0.001
Moderately or Extremely Safe	152 (75.25)	7 (7)	
Neither Safe or Unsafe	26 (12.87)	3 (3)	
Moderately or Extremely Unsafe	24 (11.88)	90 (90)	
How concerned are you that a shooting could occur at your child’s school?			<0.001
Not at all or a little concerned	108 (53.47)	1 (1)	
Moderately to Extremely concerned	94 (46.53)	99 (99)	
Would you feel safer if guns were available to teachers in schools?			
Yes	45 (22.28)	6 (6)	<0.001
No	157 (77.7)	94 (94)	
Should armed police be present in schools?			
Yes	155 (76.73)	74 (74)	0.107
No	26 (12.87)	8 (8)	
I do not know	21 (10.40)	18 (18)	
If you believe that school staff should have access to guns, who specifically should have access to the guns?			
Principal	29 (14.50)	19 (19)	0.316
Teachers	23 (11.50)	7 (7)	0.221
Security Officers	88 (44)	72 (72)	<0.001
All Staff	24 (12)	5 (5)	0.053
If you believe that school staff should have access to guns, how should guns be stored in school?			
Staff should be able to carry the guns	33 (17.28)	1 (1)	<0.001
Locked gun cases within the classroom or on school grounds	54 (28.27)	28 (28)	
Other	16 (8.38)	0 (0)	
I do not believe that school staff should have access to guns	88 (46.07)	71 (71)	
What do you think should be done to make schools safer?			
Entry points to schools and school grounds should be limited, controlled, and staffed by trained professionals	169 (84.50)	100 (100)	<0.001
Wireless panic alarms could be placed in every school	141 (70.50)	100 (100)	<0.001
Strategically placed telephones for making 911 calls set up around schools	137 (68.50)	100 (100)	<0.001
Education for students and teachers to recognize and report concerning behavior or the presence of people who do not belong in the school at all	156 (78)	99 (99)	<0.001
Metal detectors at school entrances	104 (52)	100 (100)	<0.001
Nothing—schools are safe as they are	6 (3)	5 (5)	0.385
How difficult do you believe it is for a child to get access to a gun?			
Difficult or very difficult	51 (26.15)	9 (9)	<0.001
Neither difficult nor easy	59 (30.26)	17 (17)	
Easy or very easy	85 (43.59)	74 (74)	
Does your child’s school have metal detectors?			
Yes	21 (10.61)	27 (27)	<0.001
No	167 (84.34)	10 (10)	
I do not know	10 (5.05)	63 (63)	
Do you feel that your child’s school is more or less safe than their neighborhood?			
Safer	42 (21.32)	4 (4)	<0.001
Equally as safe	134 (68.02)	19 (19)	
Less safe	21 (10.66)	77 (77)	
Do you own a gun?			
Yes	40 (20.41)	0 (0)	<0.001
No	149 (76.02)	74 (74)	
Prefer not to answer	7 (3.57)	26 (26)	
Should guns in the home be stored in a locked gun safe?			
Yes	166 (85.1)	93 (93)	0.051
No	29 (14.9)	7 (7)	
Have you ever fired a gun?			<0.001
Yes	108 (55.10)	7 (7.07)	
No or prefer not to answer	88 (44.90)	92 (92.93)	
If you have fired a gun, under what circumstances have you fired a gun?			
Military	10 (5.10)	0 (0)	<0.001
Law enforcement service	6 (3.06)	0 (0)	0.577
Self-defense	3 (1.53)	0 (0)	<0.001
Hunting	39 (19.90)	0 (0)	<0.001
Target practice or shooting range	89 (45.51)	0 (0)	<0.001
Have you ever had formal training on gun use and gun safety?			
Yes	72 (36.55)	0 (0)	<0.001
No	114 (57.87)	93 (93)	
Prefer not to answer	11 (5.58)	7 (7)	
Have you ever been a victim of gun violence?			
Yes	16 (8.12)	12 (12)	0.001
No	175 (88.83)	74 (74)	
Prefer not to answer	6 (3.05)	14 (14)	
Do you personally know anyone who has been a victim of gun violence?			
Someone in my family has been a victim of gun violence	16 (8.16)	2 (2)	<0.001
A close friend has been a victim of gun violence	16 (8.16)	11 (11)	
Both someone in my family and a close friend have been victims of gun violence	23 (11.73)	4 (4)	
No	131 (66.84)	62 (62)	
Prefer not to answer	10 (5.10)	21 (21)	

Safety concerns

While an overwhelming 75% of UNY respondents felt their child is moderate to extremely safe in school, almost half (46.5%) expressed moderate to extreme concern about shooting occurrences at their child’s school. A minority (7%) of NYC respondents felt their child is moderately to extremely safe in school and almost all NYC respondents (99%) were moderately to extremely concerned about school shooting.

Guns to ensure safety in schools

A significantly higher proportion (22.3%) of the UNY site respondents agreed they would feel safer if guns were available to teachers, compared to only 6% of the NYC respondents (p<0.001). However, almost a similar majority of respondents at both sites agreed on the presence of armed police at schools (76.73% at UNY vs 74% at NYC p=0.107). Most NYC parents (71%) did not believe that school staff should have access to guns, compared to only 46.1% of UNY parents (p<0.001). And when agreeing to school staff having access to guns, 72% of NYC parents wanted guns to be in the hands of security officers compared to 44% of the UNY who felt the same (p<0.001)

Most study respondents agreed with almost all proposed solutions for making schools safer. Such solutions included control of entry points, panic alarms, 911 telephone stations, safety education, and metal detectors. However, there were differences in agreement by site with almost 100% of NYC parents agreeing to all solutions and agreement of UNY parents ranging from as low as 52% for metal detectors to high as 85% for controlled school entry points.

Parental experience with guns

About one-fifth of UNY parents (20.4%) reported owning a gun compared to none of NYC parents (p<0.001) with a caveat that 26% of parents from NYC preferred not to answer, while 3.4% of parents from UNY chose not to answer this question. Additionally, 55.1% of UNY parents reported firing a gun in the past compared to 7.1% of NYC parents (p <0.001). No respondent from NYC had formal training on gun use and safety compared to 36.6% of UNY parents (p<0.001). Sixteen parents from UNY reported being a victim of gun violence and 12 from NYC (8.12% vs 12% p <0.001).

Tables 3-5 compare the responses of parents regarding how safe parents felt about their child at school, the shooting-related concerns, and their experience with guns.

**Table 3:** Comparison of responses of parents by how safe parents felt about their child at school

	How safe do you feel that your child is in school?			
	Moderately or Extremely Safe (N=159)	Neither Safe nor Unsafe (N=29)	Moderately or Extremely Unsafe (N=114)	P value
	n(%)	n(%)	n(%)	
Would you feel safer if guns were available to teachers in schools?				
Yes	36 (22.6)	3 (10.3)	12 (10.5)	0.019
No	123 (77.4)	26 (89.7)	102 (89.5)	
Should armed police be present in schools?				
Yes	122 (76.7)	19 (65.2)	88 (77.2)	
No	20 (12.6)	3 (10.34)	11 (9.7)	
I do not know	17 (10.7)	7 (24.1)	15 (13.2)	
If you believe that school staff should have access to guns, who specifically should have access to the guns?				
Principal	27 (17.1)	0 (0)	21 (18.6)	0.044
Teachers	21 (13.3)	0 (0)	9 (8.0)	0.059
Security Officers	75 (47.5)	10 (34.5)	75 (66.4)	0.001
All Staff	20 (12.7)	1 (3.5)	8 (7.08)	0.152

**Table 4:** Comparison of responses of parents about their concerns of shooting at school

Are you concerned that a shooting could occur at your child’s school?			
	Yes (N=193)	No (N=109)	P value
	n(%)	n(%)	
Would you feel safer if guns were available to teachers in schools?			
Yes	23 (11.9)	28 (25.7)	0.002
No	170 (88.1)	81 (74.3)	
Should armed police be present in schools?			
Yes	153 (79.3)	76 (69.7)	0.004
No	13 (6.7)	21 (19.3)	
I don’t know	27 (14.0)	12 (11.01)	
If you believe that school staff should have access to guns, who specifically should have access to the guns?			
Principal	34 (17.6)	14 (13.1)	0.305
Teachers	12 (11.2)	18 (9.3)	0.601
Security Officers	49 (45.8)	111 (57.5)	0.051
All Staff	16 (15.0)	13 (6.7)	0.021

**Table 5:** Comparison of responses of parents about their experience with guns

Do you own a gun?				
	Yes (N=40)	No (N=223)	Prefer not to answer (N=33)	P value
	n(%)	n(%)	n(%)	
Would you feel safer if guns were available to teachers in schools?				
Yes	19 (47.5)	24 (10.8)	6 (18.2)	<0.001
No	21 (52.5)	199 (89.2)	27 (81.8)	
Should armed police be present in schools?				
Yes	36 (90)	166 (74.4)	24 (72.7)	0.236
No	2 (5)	27 (12.11)	3 (9.1)	
I don’t know	2 (5)	30 (13.5)	6 (18.2)	
If you believe that school staff should have access to guns, who specifically should have access to the guns?				
Principal	8 (20.0)	32 (14.4)	7 (21.2)	0.458
Teachers	9 (22.5)	17 (7.7)	4 (12.1)	0.016
Security Officers	18 (45.0)	117 (52.7)	23 (69.7)	0.095
All Staff	9 (22.5)	13 (5.9)	6 (18.2)	0.001

Responses by how safe parents felt about their child at school

Overall, of the 159 respondents who felt their child is moderately or extremely safe at school, 22.6% felt that they would feel safer if guns were available to teachers compared to 10.5% of the 114 who felt their child was moderate to extremely unsafe at school and, 10.3% of the 29 who felt their child is neither safe nor unsafe (p=0.019). Overall, about three-fourths of parents agreed to the presence of armed police at school and the agreement did not differ significantly by how safe parents felt about their child at school (p=0.352). However, the responses did differ significantly when asked about who specifically among the school staff should have access to guns with 47.5% of those feeling safe wanting it to be security officers compared to 66.3% of those feeling unsafe about their child at school and 34.5% of those who felt neither safe nor unsafe.

Parental responses regarding concerns with school shootings

Overall, of the 193 respondents who indicated they are concerned about a shooting at their child’s school, about 12% indicated they would feel safer if guns were available to teachers in schools compared to almost twice (26%) of the 109 who were not concerned (p=0.002). There was a statistically significant difference between groups when asked if armed police should be present in school, with about 80% of parents who were concerned about a shooting stating armed police should be present compared to about 70% of those who were not concerned (p=0.004).

Parental responses regarding personal experience with guns

Overall, of the 40 parents who reported owning a gun, 47.5% felt safer if guns were available to teachers, compared to 10.8% of the 223 who did not own a gun and 18.2% of those who preferred not to answer a question about gun ownership (p<0.001). Similarly, a higher proportion of gun-owning parents (90%) preferred armed police presence at school compared to 74.4% of non-owners and 72.7% of those who preferred not to answer, although differences were not statistically significant (p=0.236).

Overall, of the 115 parents who reported ever firing a gun a significantly higher proportion (28.7%) felt safer if guns were available to teachers compared to those who never fired one or preferred not to answer (28.7% vs 8.9%, p<0.001).

## Discussion

This is one of the first studies focusing on differences in attitudes toward arming teachers between parents residing in predominantly rural/suburban (and presumably more “gun-friendly”) and urban settings. In this study, most parents reject the idea of teachers carrying or having access to guns at school. Overall, less than a quarter of parents responded by indicating they would feel safer if teachers had guns in school. Nevertheless, the perceptions of safety and the probable solutions to minimize gun violence/shootings at school differ by location and the sociodemographic composition of the respondents. To the best of our knowledge, this is the first study of its kind to survey parents on their perceptions of school firearm safety as well as their opinions on teachers carrying guns at school in both a large city as well as both suburban and urban locations. The significant difference in concern for school safety, with NYC respondents feeling much less safe in school, may be attributed in part to differences in race but is also likely related to several confounding factors including socioeconomic, cultural, and psychosocial factors with many external influences. Inherent attitudes toward guns and gun ownership are likely different in an urban inner-city environment when compared to a more suburban and rural location where hunting and shooting sports activities are more prevalent. Mowen and Freng found a difference in perception of school safety by race, specifically regarding Hispanics, among students but not among parents (national survey larger study, but results are almost 20 years old). This study by Mowen and Freng also demonstrated that parents with a child attending school in a higher crime area, greater levels of delinquency, or a school with higher portions of students receiving free or reduced lunch reported significantly lower levels of safety compared to counterparts [10]. The ED from which our NYC sample was surveyed is located in an area with high crime rates and greater poverty, which may impact the perceptions of safety and partially explain the results we have found [11,12].

As a safety measure, guns in the hands of either teachers or school staff was overwhelmingly rejected by NYC parents. However, there was some acceptance of it by UNY parents with one-fifth agreeing for teachers and two-thirds agreeing for school staff to carry guns for safety reasons. Perhaps one of the reasons for this difference is consistent with gun ownership and experience in handling guns. In UNY, 20% of respondents identified as gun owners where zero identified as gun owners in NYC (with a larger percentage of prefer not to answer). Fifty-five percent of respondents in UNY compared to only 7% in NYC reported ever firing a gun in the past. Lower gun ownership in urban neighborhoods simultaneously with higher rates of violence and crime is well known [13]. Instead, the largest demographic of gun owners in the US are White men living in rural communities who are earning more than $100K/year [14]. The UNY population in our study was predominantly White with a higher proportion living in rural and suburban areas, compared to predominantly Hispanic and 100% urban residents. Perhaps those living in the inner-city population are more likely to associate gun ownership with violence and not the prevention of violence. On the other hand, urban and suburban folks associate gun ownership with safety and recreation-hence the difference in perceptions. In our study, gun ownership was a statistically significant factor in parents reporting that they would feel safer if teachers had guns in schools. It should be noted that gun ownership has been reported as a risk factor in cases involving homicide [15]. However, in the survey performed by Wolfson et al., support for guns in schools remained less than 1/5th even among a large percentage of respondents who identified as gun owners and veterans [16]. This suggests there is likely more than just gun ownership contributing to overall opinions and preferences and multiple factors at play, or perhaps the specific opinions of parents differ from those of the general population.

Limitations

This study was limited by the self-reporting of responses which, although anonymous, may not be truly representative of gun ownership and prior experience with guns. For some questions, several respondents preferred not to answer, and the impact of these assertions on results is difficult to gauge. The survey tool also lacks external validation. Although this study has captured urban, suburban, and rural populations, it was still only conducted in the state of New York, and therefore limits opinions to one part of the country. We have also noted that there is a lack of diversity in the NYC population, containing a large percentage of Hispanic respondents. Therefore, it cannot be assessed if responses varied by race within communities. Finally, a limitation of survey administration in the ED is that the population surveyed may be biased toward sick or injured people visiting an emergency healthcare setting and do not represent the general population. Further studies may be warranted to comment on the generalizability of the opinions in other parts of the country and in other settings.

Additional studies in areas where self-reporting of gun ownership is higher are warranted to determine if the results of our survey are reproducible. Furthermore, our survey was aimed at parental opinion, but it is also important to determine the opinions of children and adolescents, insofar as we know that they are significantly impacted by violence in schools. Future studies are needed to determine whether older children and adolescents are in favor of teachers and staff carrying guns in schools as a deterrent to more violence. Gun laws vary by state and by region within the state [9]. Further studies examining parental opinions in other states with less strict gun laws would be of interest to compare to the opinions of residents in New York and NYC where there are stricter concealed carry laws in place.

Our study supports the opinions of large organizations, that arming teachers is likely not in the best interest of school safety [8]. Guns in school pose significant risks. From the public health perspective, prevention is always desired, nevertheless, there is a considerable risk involved when access to a firearm is increased. Perhaps it is also unfair to put the burden on teachers.

## Conclusions

This study found that, although the perception of child safety in schools and, reported experiences with guns varied significantly, parents in both communities largely agreed on potential solutions. Specifically, most parents agreed that it should be the security officers and not teachers who should be carrying firearms and that armed police should be present in schools to provide safety. Most parents surveyed were in favor of instituting other measures to make school safer including limiting entry points, panic alarms, strategic 911 phones, education, and metal detectors. Policymakers and government officials should focus on these areas to improve overall safety and deter gun violence. The costs and feasibility of universally instituting measures such as these are currently unknown and should also be a focus of additional research.
